# Pseudaneurysm of the superolateral genicular artery following an anterior cruciate ligament reconstruction

**DOI:** 10.1016/j.ijscr.2020.02.050

**Published:** 2020-02-28

**Authors:** L. Glanz

**Affiliations:** a Department of Orthopedic Surgery and Musculoskeletal Trauma Care, University Hospitals Geneva, Geneva, Switzerland; b Civil Hospices of Lyon, Université Claude Bernard Lyon 1, Lyon, France

**Keywords:** Anterior cruciate ligament, Complications, Pseudoaneurysm, False aneurysm, Vascular injury, Pulsatile mass

## Abstract

**Introduction:**

Arthroscopic procedures are a safe way nowadays to do anterior cruciate ligament reconstruction. Rare complications involve injuries to popliteal vessels or one of its branches.

**Presentation of case:**

We present a case of a young patient who undergone an anterior cruciate ligament reconstruction. This procedure was associated with a meniscal suture.

The follow-up was marked by pseudoaneurysm of the supero-lateral genicular artery.

**Discussion:**

This is the first time a pseudoaneurysm of this branch is described in the literature as we discovered it. The treatment by an ultrasonography-guided embolization with thrombin was proceeded and suceeded.

**Conclusion:**

We stress the fact with new arthroscopic procedures and techniques, new complications can occur, and we should be attentive to them and new symptoms consequently, as a painless mass on the lateral side of the knee, to ensure a fast and optimal treatment.

## Highlights

• Pseudoaneurysm of the superolateral genicular artery is a risk in arthroscopic knee procedure.
• Pseudoaneurysms risk increase due to minimal invasive procedure.
• Ultrasound-guided percutaneous thrombin injection for iatrogenic pseudoaneurysms is a valuable option.

The case report has been reported in line with the SCARE criteria [1].

## 1 Introduction

Intraoperative complications of primary anterior cruciate ligament (ACL) reconstruction surgery are rare and mostly relied to tunnel placement, conflict between the tendon graft and the bone reliefs, arthrofibrosis mostly as « cyclop syndrome », fixation failure [2], and consequences of the previous ones as posterior wall « blow-out » fracture or graft rupture due to friction against bone relief.

Vascular injuries are a rare complication of arthroscopic procedure, and mostly described for the popliteal vessels.

The present case reports, as first time described in the literature, a pseudoaneurysm of the supero-lateral genicular artery following an ACL reconstruction with bone-quadriceps-tendon autograft and an “outside -in” technique for the femoral tunnel.

## 2 Presentation of case

A 17 years old patient, without comorbidities, had sustained a valgus mechanism trauma on his right knee playing tennis resulting in an acute complete ACL tear.

Two months after the trauma, an arthroscopic anterior cruciate ligament reconstruction procedure was performed using a bone-tendon autograft consisting of a quadricipital tendon.

The technique applied has been previously described [3] using two tunnels placements, one on the tibial side and one on the external femoral side.

For the femoral tunnel, an outside-in aiming device is used. The lateral femoral tunnel is defined by device, performing a stab-incision.

The autograft is fixed with two absorbable screws, one in each tunnel.

Meniscal suture for a meniscal tear was also supplied during the arthroscopic time of surgery. It was done with an « all-inside » suture anchor.

The procedure was all done without any peculiar intra-operative problem.

The patient was discharged from the hospital, 3 days after surgery.

There were no sensory loss nor pulse deficit with an intact neuro-vascular status. He was allowed to perform partial weight bearing with crutches, as a routine manner in the department because of the meniscal suture and 0° to 90° flexion from extension range of motion.

During the routine clinical follow-up, the patient presented on the 19th day of surgery with a pulsatile painless mass on the external side of the knee. He did not complain neither about any pain or limited range of motion of the knee in the few past days before.

No clinical evidence of site infection was noted.

**Fig. 1:**
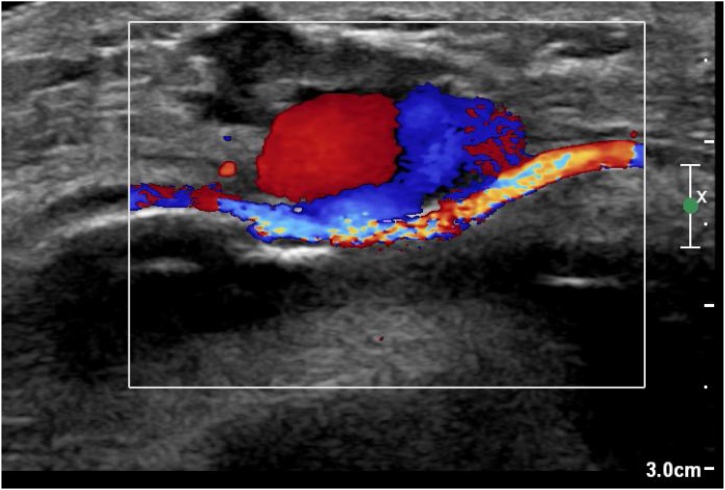
Ultrasonogrpahy showing the pulsatile mass with aliasing flow inside and the continuity with the artery with a narrow neck.

**Fig. 2:**
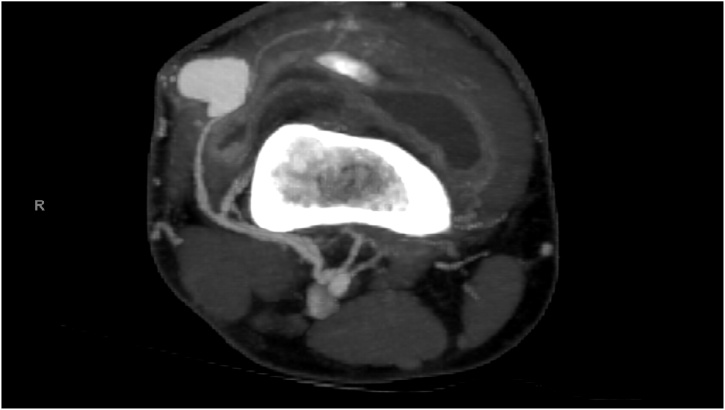
CTA showing the pseudoaneurysm. There is still a little edema due to the procedure. Axial plane of CTA showing the early opacification of pseudoaneurysm with contrast in arterial phase. Note the synovitis in the knee joint (post-operative aspect).

**Fig. 3:**
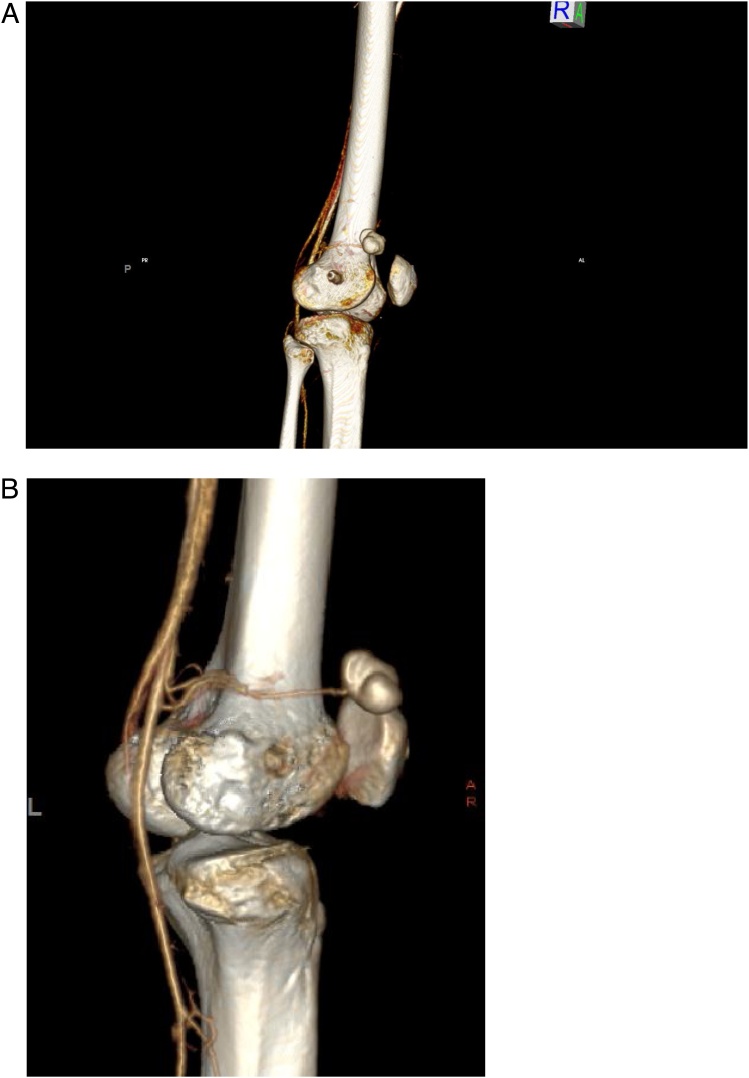
A Lateral view of a 3D Reconstructed CT with the visualization of the pseudoaneurysm depending of the supero-lateral genicular artery. B ¾ view with the popliteal artery and the pseudoaneurysm of its branch.

A Doppler Ultrasonography was performed suggesting a vascular pseudo aneurysm on the antero- external side of the knee (Fig. 1). A computed tomography angiography (CTA) completed, confirmed a pseudo aneurysm with an arterial feeder from the supero-lateral genicular artery (a popliteal artery branch) (Figs. 2, 3A–B).

Thus, an ultrasonography-guided embolization with thrombin was proceeded. Prophylactic thrombosis by enoxaparine was then continued 4 h after the procedure as a usual routine for ACL reconstruction in our department.

An ultrasonography was programmed 24 h after the embolization as a control manner.

The long-term follow-up at 1 year demonstrated a good evolution as for the ACL reconstruction and the meniscal suture, with no edema, complete flexion and extension and no signs of infection, as for the resolution of the pseudoaneurysm.

The patient was completely satisfied with these procedures.

## 3 Discussion

This is the first report of a pseudoaneurysm of the supero-lateral genicular artery after a primary ACL reconstruction using an outside-in technique for the femoral tunnel and a quadriceps tendon autograft.

True aneurysms are a dilation of the artery including its three layers, while pseudoaneurysms involve dilation of an artery with actual disruption of one or more layers of its walls. The etiology of true and false aneurysms also differs, The latter being mostly traumatic in contrast of degenerative true ones [4,5].

The incidence of iatrogenic pseudo-aneurysm has risen these last years [6] with probably a contribution by demand of minimal invasive procedures [4].

Pseudo-aneurysm, false aneurysm, are the major periprocedural complications secondary to vascular interventions. They can be traumatic or following infections too.

Obesity, hypertension and calcified arteries are ones of predisposing factors that are not present to this patient [7].

Knee arthroscopy is a safe procedure with less than 1% of complications [8].

Vascular complications are uncommon and are indeed subjects to case reports [[9], [10], [11]]. Moreover, false aneurysm should be considered at risk of rupture [13,14].

Several cases reports are about popliteal artery pseudo-aneurysm [12,11] but none with a supero-lateral genicular artery.

Performing the femoral tunnel with the aiming device through a minimal skin incision technique may put the artery and its branches at risk. Moreover, false aneurysm should be considered at risk of rupture [13,14].

Although there is no evaluation of this risk for the genicular artery, it was estimated [7] at 24% in femoral ones leading to limb and life threatening in 12%. This is mostly after cardiac and vascular catherization.

Concerning the treatment chosen in this presented case, ultrasound-guided percutaneous thrombin injection repair for iatrogenic pseudoaneurysms was initially introduced by Liau and associates [15] in 1997.

This technique is mostly considered for false aneursyms with a narrow neck to avoid leakage at the target embolization. It has become the first line treatment in many centers due to its success rate of 91–100% and a low rate of 2 percent of complications [7].

Conservative treatment is also possible for false aneurysm less than 3 cm in diameter [16] but it seems that delayed presentation more than 2 weeks after surgery or the use of anticoagulation are indicative of a non-spontaneous regression [17,7].

Taking all of these, we should be careful about percutaneous aiming device we use routinely for example in elective arthroscopic procedures or, in other way, in trauma for closed reduction and internal fixation.

Even knowing the precise anatomy of the knee and its vessels, such genicular arteries are difficult to see per-operatively.

Although the non-systematic use of a tourniquet, it can be suggested to deflate it before the end of the procedure and taking care of any bleeding in these areas of even small skin incisions, as we know the possibility of injury to this type of vascular components.

Furthermore, it is the first case noticed in our hospital among hundreds ACL reconstruction per year using this technique.

## 4 Conclusion

As a first time described in the literature, a pseudoaneurysm of the supero-lateral genicular artery following an ACL reconstruction can have good response of a treatment algorithm used for others known traumatic aneurysms.

We also stress the fact, this is another new example that new arthroscopic procedures as new techniques can have new complications never described before or we never thought about accordingly.

This case suggests that a painless mass of the knee, on the external side for example, even in a young healthy patient, can be the first or only symptom of a vascular injury.

We should take this into account for further ACL reconstructions with percutaneous devices to avoid it and be attentive during the follow-up. This new knowledge leading to a fast diagnosis with a good and optimal result without any sequelae.

## Funding

No sponsor.

## Ethical approval

No need of ethical approval in this case report in agreement with the reasons mentioned above.

## Consent

The patient in this case report gave his consent.

## Author contribution

Ludovic Glanz: I wrote the entire paper, collected data, decided to publish, take decision for this paper, validation and visualization.

## Research studies

This is not a human study, but a case report.

## Guarantor

Ludovic Glanz.

## Provenance and peer review

Not commissioned, externally peer-reviewed.

## Declaration of Competing Interest

Nothing to declare.
